# Intestinal bacteria-derived extracellular vesicles in metabolic dysfunction-associated steatotic liver disease: From mechanisms to therapeutics

**DOI:** 10.1016/j.mocell.2025.100216

**Published:** 2025-04-14

**Authors:** Li-Na Qin, Yun-Feng Yu, Lie Ma, Rong Yu

**Affiliations:** 1 Department of Endocrinology, The First Hospital of Hunan University of Chinese Medicine, Changsha, Hunan, China; 2 Department of Reproductive Medicine, The Third Affiliated Hospital of Henan University of Traditional Chinese Medicine, Zhengzhou, Henan, China; 3 College of Chinese Medicine, Hunan University of Chinese Medicine, Changsha, Hunan, China

**Keywords:** Bacteria-derived extracellular vesicle, Intestinal microbiota, Metabolic dysfunction-associated steatotic liver disease, Nonalcoholic fatty liver disease, Therapeutic applications

## Abstract

Metabolic dysfunction-associated steatotic liver disease (MASLD) is a progressive disease that affects the health of approximately one-third of the world's population. It is the primary cause of end-stage liver disease, liver malignancy, and liver transplantation, resulting in a great medical burden. No medications have yet been approved by the US Food and Drug Administration for treating MASLD without liver inflammation or scarring. Therefore, the development of specific drugs to treat MASLD remains a key task in the ongoing research objective. Extracellular vesicles (EVs) play an important role in the communication between organs, tissues, and cells. Recent studies have found that intestinal microbiota are closely related to the pathogenesis and progression of MASLD. EVs produced by bacteria (BEVs) play an indispensable role in this process. Thus, this study provides a new direction for MASLD treatment. However, the mechanism by which BEVs affect MASLD remains unclear. Therefore, this study investigated the influence and function of intestinal microbiota in MASLD. Additionally, we focus on the research progress of BEVs in recent years and explain the relationship between BEVs and MASLD from the perspectives of glucose and lipid metabolism, immune responses, and intestinal homeostasis. Finally, we summarized the potential therapeutic value of BEVs and EVs from other sources, such as adipocytes, immunocytes, stem cells, and plants.

## HIGHLIGHTS

• Gut microbiota mediate metabolic dysfunction-associated steatotic liver disease.
• Gut bacteria-hepatocyte crosstalk via bacterial extracellular vesicles.
• Bacterial and other extracellular vesicles emerge as novel therapies.

## Graphical abstract

**Figure:**
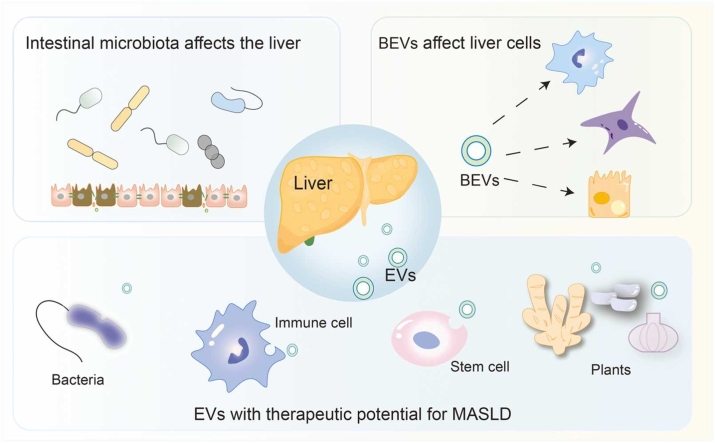

## INTRODUCTION

Nonalcoholic fatty liver disease (NAFLD) is a chronic disease characterized by abnormal fat accumulation in hepatocytes. It involves multiple systemic lesions that are significantly associated with diabetes, obesity, metabolic syndrome, and other diseases. Fat toxicity, intestinal microbiota, insulin resistance (IR), and inflammation are intertwined to form a “multiple hit” to the liver on multiple levels (Tilg et al., 2021). In 2020, the term NAFLD was revised to metabolic associated fatty liver disease followed by subsequent refinement in 2023 to metabolic dysfunction-associated steatotic liver disease (MASLD) through an international multisociety Delphi consensus initiative (Rinella et al., 2023). This new definition emphasizes the phenomenon that the disease often coexists with other metabolic diseases, such as overweight, obesity, and type 2 diabetes (T2D), for example, more than 70% of T2D patients are complicated with NAFLD (Eslam et al., 2020a, Eslam et al., 2020b, Tilg et al., 2017). To enhance terminological consistency throughout our discourse, we will systematically employ the updated MASLD nomenclature as the unifying designation encompassing previous NAFLD/metabolic associated fatty liver disease classifications, whereas nonalcoholic steatohepatitis will be referred to as metabolic dysfunction-associated steatohepatitis (MASH). Currently, it is the most common liver disease in western countries and has shown a rapid growth trend in developing countries, affecting 30% of the global population (Miao et al., 2024). Although simple steatosis is generally not associated with adverse clinical outcomes, a cohort study revealed that patients with MASLD demonstrated significantly higher risks of extrahepatic malignancies and liver-related mortality compared to non-MASLD populations (Simon et al., 2021). Furthermore, patients with MASH and stage ≥2 hepatic fibrosis have significantly increased morbidity and mortality due to liver-related conditions (Newsome et al., 2020). As of March 2024, the US Food and Drug Administration approved Rezdiffra (resmetirom) as the first medication for treating MASH, a severe form of fatty liver disease (Chen et al., 2025). However, there are currently no Food and Drug Administration-approved medications for treating MASLD without liver inflammation or scarring. Therefore, there is an urgent need to further explore the pathogenesis of MASLD and develop appropriate drugs.

Extracellular vesicles (EVs) have a vesicular structure consisting of double lipid membranes and contain active substances such as lipids, proteins, and genetic material (Takahashi and Takakura, 2023), reflecting the characteristics and identity of the source cells. EVs are widely distributed in a variety of body fluids (feces, blood, ascites, etc), and stressed or normal cells can release EVs outside of the cell (Thietart and Rautou, 2020). The intestinal microbiota plays a key role in glucose and lipid metabolism, and the regulation of liver function, thus affecting the progression of MASLD. The bacteria-derived extracellular vesicles (BEVs) released by bacteria in feces can enter liver cells (Villard et al., 2021). BEVs have the potential for interorgan metabolic communication, mediate intercellular information exchange in the intestine-liver axis, and participate in various physiological and pathological processes in the body (Takahashi and Takakura, 2023). Both eukaryotic- and bacterial-derived EVs can enter hepatocytes and affect the progression of MASLD (Villard et al., 2021). Therefore, BEVs are promising diagnostic tools and treatments.

Therefore, in this review, we focus on the mechanism of action of intestinal microorganisms, especially BEVs produced by them, in the occurrence and development of MASLD. Additionally, we summarized the roles and mechanisms of other related EVs in MASLD.

## INTESTINAL MICROBIOTA ARE INVOLVED IN MASLD

There is a complex and large microbial community in the human intestinal tract, containing more than 10 trillion microorganisms (Leung et al., 2016). The metabolites (toxic factors and nutrients) produced by these microorganisms are transported to the liver through the portal vein, thereby affecting liver pathophysiology. The liver is an important metabolic organ that converts and synthesizes substances, such as bile acids, cholesterol, and proteins, which are transported to the intestinal tract through the bile duct and absorbed and utilized by intestinal cells and microbiota. Therefore, there is close bidirectional crosstalk between the intestine and liver.

**Fig. 1:**
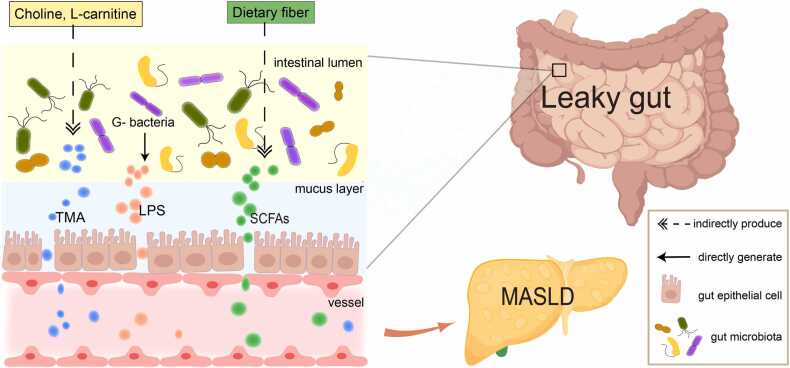
Intestinal microbiota mediates pathological changes in MASLD. TMA, trimethylamine; LPS, lipopolysaccharide; SCFAs, short-chain fatty acids; MASLD, metabolic dysfunction-associated steatotic liver disease.

MASLD is closely associated with an imbalance in intestinal microbiota. Microbiota imbalance increases intestinal permeability to harmful substances and liver exposure to intestinal metabolites (Leung et al., 2016). Studies have shown that a decrease in intestinal microbiota diversity, a decrease in beneficial microbiota, and an increase in bacterial pathogens lead to the occurrence and progression of MASLD, which is related to its severity. According to previous reports, compared with the matched control group, in patients with MASLD, the abundance of *Bacteroidetes* decreased, whereas the abundance of *Firmicutes* and *Proteobacteria* increased, and significant changes occurred at the family and genus levels (Boursier et al., 2016, Zhang et al., 2022). Dendritic cells (DCs) engulf antigens in Peyer's patches, limiting the immune response to the mesenteric lymph nodes; however, if the intestinal barrier is damaged, the bacterial component can pass through the portal vein into the liver (Balmer et al., 2014, Tilg et al., 2020), leading to increased microbial metabolites in the systemic circulation (Chen and Vitetta, 2020). Therefore, a systemic immune response consistent with parenteral commensal bacterial exposure was observed in patients with MASLD (Balmer et al., 2014). Bacteria ferment soluble dietary fibers to produce short-chain fatty acids (SCFAs), including acetic, butyric, and propionic acids (van der Hee and Wells, 2021). SCFAs can induce the release of glucagon-like peptide 1 (GLP-1) and peptide YY by activating the G-protein-coupled receptors (GPR41 and GPR43) of endocrine L-cells located in the intestinal tract, leading to increased insulin secretion and delayed gastric emptying, thereby promoting fatty acid oxidation and reducing fat accumulation (Zhang et al., 2023). Lipopolysaccharide (LPS), a key structural component of the outer membrane in Gram-negative bacteria, is predominantly released through bacterial cell lysis. The majority of LPS originates from members of the phylum Bacteroidetes (Di Lorenzo et al., 2019, Hersoug et al., 2016). Patients with lean MASLD have endotoxemia, and LPS stimulates macrophages to release inflammatory factors such as tumor necrosis factor α (TNF-α) and interferon-γ by binding with Toll-like receptors (TLR), thus damaging the metabolic adaptability of the human body (Alharthi et al., 2023). Trimethylamine (TMA) is primarily generated through gut microbial metabolism of dietary substrates rich in carnitine and choline (Dalla Via et al., 2020, Tang et al., 2013). TMA is transformed into trimethylamine N-oxide by the liver flavin monooxygenases. Trimethylamine N-oxide activates the inflammatory corpuscles containing pyrin domain 3 (NLRP3), thereby inducing oxidative stress damage in hepatocytes (Rodrigues et al., 2024) (Fig. 1).

A study (Le Roy et al., 2013) transplanted fecal microbiota from obese mice with or without MASLD into mice fed a high-fat diet (HFD), and both groups became obese. However, mice transplanted with MASLD bacteria showed elevated blood sugar levels, IR, and fatty liver degeneration. Another investigation (Craven et al., 2020) transplanted intestinal microbiota from a thin donor into patients with MASLD, which improved the intestinal barrier function of the patients, reduced the proportion of liver steatosis, and ameliorated IR. Additionally, further research (Soderborg et al., 2018) transplanted the intestinal microbiota of 2-week-old infants born to obese or normal-weight mothers into germ-free mice. Their findings revealed that fecal microbiota transplantation from obese mothers induced intestinal barrier dysfunction in recipient mice, characterized by suppressed gene expression of tight junction proteins and elevated intestinal permeability. Furthermore, microbial transfer results in impaired macrophage phagocytic activity and upregulates inflammatory markers in the portal vein (Soderborg et al., 2018). In conclusion, an imbalance in the intestinal microbiota is an important risk factor for host MASLD.

## BEVS FROM INTESTINAL BACTERIA

Intestinal microorganisms usually remain in the intestinal lumen, therefore, it is difficult for them to interact directly with the host organs. Growing evidence suggests that BEVs may serve as biomolecular transport systems (Bittel et al., 2021). Fecal-derived EVs (fEVs) include BEVs and host eukaryotic cell-derived EVs. The human intestinal tract contains approximately 10^14^ BEVs (Tulkens et al., 2020), with sizes ranging from 20 to 400 nm (Toyofuku et al., 2019). Gram-positive and Gram-negative bacteria release BEVs into feces in various ways. Gram-negative bacteria mainly bud off through the outer membrane to form outer membrane vesicles (OMVs) (Kim et al., 2015), and produce explosive outer membrane vesicles (EOMVs) and outer inner membrane vesicles (OIMVs) through explosive cell lysis (Toyofuku et al., 2019, Tulkens et al., 2020). All BEVs contain bacterial-specific proteins. OMVs are enriched with outer membrane proteins (eg, OmpA and OmpC) and periplasmic proteins (eg, alkaline phosphatase) and are characterized by the inner leaflet of phospholipids and the outer leaflets of LPS (Volgers et al., 2018). OMVs are loaded with a large amount of hydrolase and tend to select acidic proteins, which helps ensure the nutrients of the entire bacterial community (Elhenawy et al., 2014). In Gram-positive bacteria, most BEVs are released during localized cell wall lysis, some within peptidoglycan layers, and a minority are released via explosive lysis. The BEVs of Gram-positive bacteria are characterized by surface lipoteichoic acid (Jeong et al., 2022). BEVs can induce changes in the microenvironment and facilitate information exchange between the host and the bacteria.

Mammalian host cells internalize BEVs through distinct pathways; phagocytes utilize caveolin-mediated phagocytosis, whereas nonphagocytic cells employ clathrin-dependent endocytosis, macropinocytosis, or lipid raft mechanisms (Ñahui Palomino et al., 2021, O’donoghue and Krachler, 2016). Compared with the lean control group, the total fEVs of HFD-induced obese mice decreased, whereas the level of LPS^+^ BEVs, which are BEVs specifically released by Gram-negative bacteria, increased (Jain et al., 2024). Concurrently, the level of LPS^+^ BEVs increased markedly in both hepatic tissue and portal vein plasma of obese mice. OMVs have been found in human tissues and body fluids (Craven et al., 1980, Fiocca et al., 1999). Evidence that OMVs can migrate away from their parent bacteria and enter systemic circulation, where they transfer biomolecules to distant organs of the host. In the liver, OMVs accumulate in the portal triad and are ingested by hepatocytes (Bittel et al., 2021). Studies have shown that fEVs released by the intestinal epithelium are primarily transported to the liver, followed by adipose tissue and muscle (Kumar et al., 2021). Transcellular transport or intestinal leakage-mediated translocation of fEVs to the liver occurs via the portal system. Cohort analyses revealed significantly stronger associations of serum-, urine-, and feces-derived EVs in patients with T2D than in healthy controls (Nah et al., 2019), indicating the systemic circulation of BEVs across bodily fluids. BEVs serve as vectors for bioactive cargo, including LPS, DNA, RNA, and other virus-related factors (Lee et al., 2007). In summary, BEVs may be the key mechanism by which intestinal microbiota regulate glucose and lipid metabolism.

## MECHANISMS AND FUNCTIONS OF BEVS IN MASLD

### BEVs Are Involved in Glucose and Lipid Disorders

As interdependent drivers, lipotoxicity and IR collectively promote MASLD progression through reciprocal potentiation (Pal and Méndez-Sánchez, 2023). Liver fat content strongly predicts IR and precedes the appearance of adipocyte hypertrophy, cell death, and inflammation (Meex and Watt, 2017, Strissel et al., 2007). When IR occurs in the body, triglyceride decomposition in adipose tissue increases, and free fatty acids (FFAs) are released into the bloodstream. These FFAs are subsequently transported to the liver, where they undergo re-esterification into triglycerides, resulting in increased lipid droplet deposition in the liver (Smith et al., 2020). Moreover, FFAs activate inflammatory pathways in the liver (Mendez-Sanchez et al., 2018). The production of toxic lipids, such as diglycerides and ceramides, increases, exacerbating inflammation and liver injury, thereby completing a series of pathogenic processes (Perla et al., 2017;  Table 1).

**Table 1:** Summary of the mechanisms and functions of BEVs

Bacterial species/BEV source	Disease contexts/model	Key mechanisms	Effect	References
Feces of patients with T2D or mice fed with HFD	Lean mice, germ-free mice	PC of BEVs ↑, IRS2/PI3K/Akt ↓	Harmful	(Kumar et al., 2021)
*Helicobacter pylori*	HepG2 cells	IRS1/AKT-2/GLUT2 ↓	Harmful	(Talebi et al., 2024)
Intestinal microbiota	ASC−/− mice fed with HFD	HMGB1↑; aggravate hepatic steatosis	Harmful	(Chen et al., 2019)
Intestinal microbiota	Diet-induced obese mice	TLR-4↑, chemokines↑, F4/80, CD86, and CD206 ↑	Harmful	(Jain et al., 2024)
Purified fEVs from wild mice and healthy humans	Wild-type C57BL/6 mice	Increased levels of TNF-α and IL-6 through the TLR-2 or TLR-4 pathway	Harmful	(Park et al., 2018)
*E. coli*	Wild-type C57BL/6 mice	Activate TLR-4, combined with TRIF, activate CASP11	Harmful	(Gu et al., 2019)
Feces of NASH patients	Hepatic stellate (LX cells)	G-CSF, GM-CSF, and MIF ↑	Harmful	(Fizanne et al., 2023)
*Akkermansia muciniphila*	Male C57BL/6 mice	Inflammatory factors and TLR-4 ↓, IL-10↑	Beneficial	(Ashrafian et al., 2019)
*EcN*	Caco-2/PBMCs	IL-22 and hBD-2 ↑, IL-12 and TGF-β ↓	Beneficial	(Fábrega et al., 2016)
*Lactic acid bacteria*	Alcohol-induced liver injury in mice	Nrf-2 and HO-1 ↓	Beneficial	(Jiao et al., 2025)
*Lactobacillus casei* and *Lactobacillus plantarum*	Macrophages	IL-10 ↑, TNF-α ↓	Beneficial	(Müller et al., 2021)
*Pediococcus pentosaceus*	Hepatic fibrosis induced by CCl4 in mice	Collagen accumulation and α-SMA ↓	Beneficial	(Alpdundar Bulut et al., 2020)
*Bacteroides fragilis*	DCs	IL-10 ↑, TNF-α ↓; promote Th17 cells proliferation	Beneficial	(Shen et al., 2012)
EcN and ECOR63	DCs	Th1 polarization ↑	Beneficial	(Diaz-Garrido et al., 2019)
*Lactobacillus rhamnosus* JB-1	DCs	Treg amplification ↑; TNF-a ↓	Beneficial	(Al-Nedawi et al., 2015)
Pathogenic bacteria (LPS+ BEVs)	Intestinal barrier disorders	LPS+ BEVs and PAMPs ↑	Harmful	(Tulkens et al., 2020)
*Fusobacterium nucleatum*	Macrophage/Caco-2 cocultures	RIPK1/RIPK3 ↑	Harmful	(Liu et al., 2021)
*Campylobacter jejuni*	Intestinal epithelial cells	Proteolytic activity cleaves E-cadherin and clostridial protein	Harmful	(Elmi et al., 2016)
*Helicobacter pylori*	Caco-2 cells	Disrupt tight junction protein, and alter histone H1-ATP binding to affect host gene transcription	Harmful	(Turkina et al., 2015)
EcN and ECOR63	Caco-2 cells	ZO-1 and claudin-14 ↑	Beneficial	(Alvarez et al., 2016)
EcN and ECOR63	Caco-2 cells	Claudin-14 and occludin ↑	Beneficial	(Alvarez et al., 2019)
*Akkermansia muciniphila*	Obesity and T2D mice	Activate AMPK pathway and occludin ↑	Beneficial	(Chelakkot et al., 2018)
*Akkermansia muciniphila*	Inflammatory bowel disease in mice	Mediate colonic immune regulation and intestinal barrier function	Beneficial	(Kang et al., 2013)
*Lactobacillus rhamnosus*	Alcohol-induced fatty liver in mice	Activate the AhR-IL-22-Reg3 signaling pathway	Beneficial	(Gu et al., 2021)

BEVs, bacterial extracellular vesicles; T2D, type 2 diabetes; HFD, high-fat diet; PC, phosphatidylcholine; IRS2, insulin receptor substrate 2; PI3K, phosphatidylinositol-3-kinase; Akt, protein kinase B; GLUT2, glucose transporter 2; HMGB1, high-mobility group box 1; TLR-4, Toll-like receptor 4; F4/80, adhesion G-protein-coupled receptor E1; CD86, cluster of differentiation 86; CD206, mannose receptor; TNF-α, tumor necrosis factor α; IL-6, interleukin 6; TLR-2, Toll-like receptor 2; TRIF, TIR domain-containing adapter-inducing interferon-β; CASP11, caspase-11; G-CSF, granulocyte colony-stimulating factor; GM-CSF, granulocyte-macrophage colony-stimulating factor; MIF, macrophage migration inhibitory factor; IL-10, interleukin 10; hBD-2, human beta-defensin-2; TGF-β, transforming growth factor β; Nrf-2, nuclear factor erythroid 2-related factor 2; HO-1, heme oxygenase 1; α-SMA, α-smooth muscle actin; DCs, dendritic cells; Th17, T helper 17 cells; Th1, T helper 1 cells; Treg, tegulatory T cells; LPS, lipopolysaccharide; PAMPs, pathogen-associated molecular patterns; RIPK1/RIPK3, receptor-interacting protein kinase 1/3; E-cadherin, epithelial cadherin; ZO-1, zonula occludens-1; AMPK, AMP-activated protein kinase; AhR, aryl hydrocarbon receptor; IL-22, interleukin 22; Reg3, regenerating islet-derived protein 3; EcN, *Escherichia coli* Nissle 1917; PBMCs, peripheral blood mononuclear cells; NASH, nonalcoholic steatohepatitis; CCl4, carbon tetrachloride; *E. coli, Escherichia coli*.

**Fig. 2:**
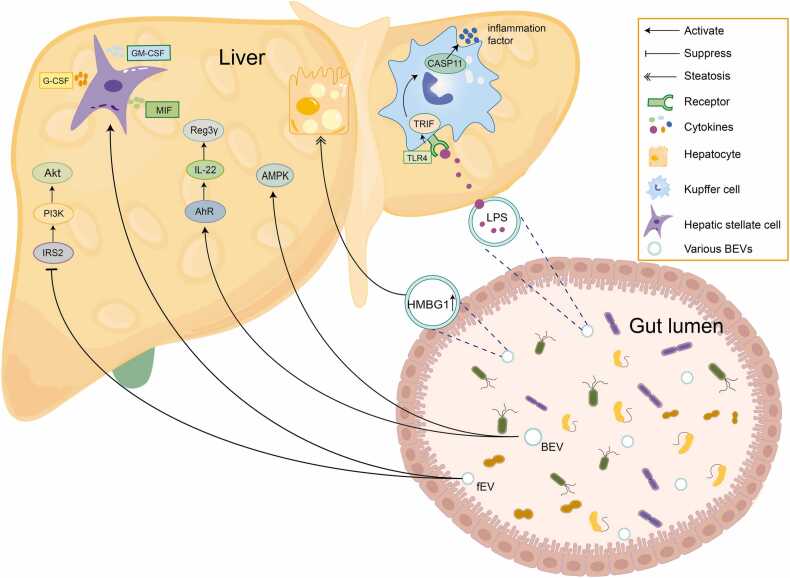
Fecal-derived extracellular vesicles affect liver cells in MASLD. G-CSF, granulocyte colony-stimulating factor; GM-CSF, granulocyte-macrophage colony-stimulating factor; MIF, macrophage migration inhibitory factor; AKT, serine/threonine-protein kinase; PI3K, phosphatidylinositol-3-kinase; IRS2, insulin receptor substrate 2; Reg3γ, regenerating islet-derived protein 3γ; IL-22, interleukin 22; AhR, aryl hydrocarbon receptor; AMPK, adenosine monophosphate (AMP)-activated protein kinase; CASP11, cysteine-aspartic protease 11; TRIF, TIR domain-containing adapter-inducing interferon-β; TLR-4, Toll-like receptor 4; HMGB1, high-mobility group box 1; LPS, lipopolysaccharide; fEV, fecal-derived extracellular vesicles; BEV, bacterial extracellular vesicles.

Administration of an HFD to lean mice significantly alters the fEV composition, characterized by reduced phosphatidylethanolamine (PE) and elevated phosphatidylcholine levels (Kumar et al., 2021). The aryl hydrocarbon receptor (AhR) is a ligand-dependent transcription factor responsible for integrating signal-initiating ligands from the environment and microorganisms (Iyer et al., 2018). Mechanistically, phosphatidylcholine-enriched fEVs from T2D models exacerbate IR through direct hepatocyte AhR binding/activation, independent of microbiota mediation (Kumar et al., 2021). In obese mice, fEVs promote hepatic IR by suppressing the expression of the insulin receptor substrate (IRS)-2, phosphatidylinositol-3-kinase (PI3K), and serine/threonine-protein kinase (AKT) (Kumar et al., 2021). The complement receptor of the immunoglobulin superfamily (CRIg^+^) macrophages clears circulating bacteria and toxins. Obesity reduces the number of CRIg^+^ macrophages, resulting in the ineffective clearance of BEVs, which in turn triggers IR and inflammation in distant tissues (Luo et al., 2021). fEVs extracted from HFD-fed mice can penetrate the intestinal barrier and be distributed to the target insulin tissues, causing IR in the liver, skeletal muscle, and adipose tissue (Kumar et al., 2021). BEVs from *Helicobacter pylori* have been shown to downregulate insulin signaling pathways, including the expression of IRS1, AKT-2, and glucose transporter 2, and induce IR in the HepG2 human hepatocarcinoma cell line (Talebi et al., 2024). Moreover, LPS-containing BEVs produced by *Pseudomonas aeruginosa* mediate the dysregulation of glucose metabolism (Choi et al., 2015). The apoptotic-related speckled protein-deficient (ASC^−^/^−^) mice intestinal microbiota is dysfunctional after being fed with HFD, the expression of high-mobility group box 1 in dysfunctional microorganism-derived BEVs is significantly increased, and high-mobility group box 1 is transported to hepatocytes by BEVs, which may be the key factor for inducing hepatic steatosis (Chen et al., 2019) (Fig. 2).

### BEVs Mediate Hepatic Inflammatory and Immune Responses

BEVs affect MASLD progression in a bacterial strain-dependent manner. The progression of MASLD to MASH is characterized by the activation of the immune system and inflammatory cells (Barreby et al., 2022). A cohort analysis found significantly increased levels of inflammatory proteins in circulating EVs in diabetic patients compared to those in the normoglycemic population, inducing endothelial cells to form plate-like pseudopodia and enhancing the migration of endothelial cells (Wu et al., 2020). Notably, BEVs from obese mice enter the liver more frequently, and the expression of Toll-like receptor 4 (TLR-4), chemokines, chemokine receptors, and macrophage markers (F4/80, CD86, and CD206) in the liver increases synchronously (Jain et al., 2024). The purified fEVs from wild mice and healthy people increased the levels of TNF-α and IL-6 through the TLR-2 or TLR-4 pathway, resulting in circulatory system inflammation (Park et al., 2018). BEVs are thought to have microbial-associated molecular patterns, which are recognized by pattern recognition receptors on cell surfaces and then trigger the release of inflammatory factors (Zhu et al., 2023). BEVs released by *Escherichia coli* (*E. coli*) carry LPS and enter hepatocytes through the portal vein to activate TLR-4. Subsequently, macrophages allow LPS in BEVs to be transported into the cytoplasm through TIR domain-containing adapter-inducing interferon-β, then activating cysteine-aspartic protease 11 to induce an immune response (Gu et al., 2019). BEVs from patients with MASH were used to interfere with the hepatic stellate (LX cells), which stimulates the release of granulocyte colony-stimulating factor, granulocyte-macrophage colony-stimulating factor, and macrophage migration inhibitory factor (Fizanne et al., 2023). An impaired gut barrier leads to leakage of BEVs, which are transferred from the intestine to the liver and absorbed by hepatocytes and hepatic stellate cells (HSCs), triggering hepatic inflammation and fibrogenic activation (Luo et al., 2022). Therefore, BEVs are likely to induce or enhance inflammation in the body (Fig. 2).

In contrast, BEVs derived from certain bacteria show positive effects. First, BEVs of *Akkermansia muciniphila* reduce the expression of inflammatory factors and TLR-4 in adipose tissue and significantly increase the expression of IL-10, an anti-inflammatory factor (Ashrafian et al., 2019). Second, *Escherichia coli* Nissle 1917 (EcN) is a probiotic strain. When EcN BEVs were cocultured with human colon adenocarcinoma cells or peripheral blood mononuclear cells, the expression of defense responses and immunoregulation-related genes increased. These results indicate that BEVs can penetrate the physical barrier of the intestinal epithelium and trigger an immune response in peripheral blood mononuclear cells. BEVs of EcN also activate the expression of the intestinal barrier factor IL-22 and antibacterial peptide β-defensin-2 and reduce the expression of IL-12 and TGF-β (Fábrega et al., 2016). Third, BEVs from *lactic acid bacteria* have been shown to reduce alcohol-induced liver inflammation and oxidative stress by downregulating the expression of Nrf-2 and HO-1 and alleviating liver tissue damage and functional damage (Jiao et al., 2025). The BEVs derived from *Lactobacillus casei* and *Lactobacillus plantarum* increase IL-10 release and reduce TNF-α release in macrophage inflammation models, and their anti-inflammatory effects are enhanced after pH optimization, stirring, and oxidative supply (Müller et al., 2021). The BEVs produced by *Pediococcus pentosaceus*, a human lactic acid commensal bacterium, may rely on TLR-2 to promote bone marrow-derived inhibitory cell differentiation and M2-type macrophage differentiation and inhibit T-cell activation. Moreover, the BEVs of *Pediococcus pentosaceus* decreased collagen accumulation and α-SMA expression in mice liver fibrosis induced by carbon tetrachloride (Alpdundar Bulut et al., 2020). Finally, the BEVs isolated from *Bacteroides fragilis* are recognized by TLR-2 of DCs by releasing capsular polysaccharide, and then are swallowed up by DCs, thus inducing an increase in IL-10 and a decrease in TNF-α (Shen et al., 2012). Additionally, BEVs affect the differentiation of T-cell subsets in a strain-dependent manner. Under the stimulation of antigen presentation and cytokines, CD4^+^ T cells can differentiate into regulatory T (Treg) and effector T cells (Th1, Th2, and Th17) (Fang et al., 2018). BEVs drive helper T- (Th) cell differentiation by affecting the release of inflammatory factors. EcN and commensal *E. coli* strains (ECOR63) BEVs induced DCs to secrete more Th1 polarization-related factors. However, commensal ECOR12 cells secrete more Treg polarization-related factors (Diaz-Garrido et al., 2019). The BEVs extracted from *Lactobacillus rhamnosus* JB-1 were gavaged in mice, which led to the amplification of Treg cells and decreased TNF-α release. Additionally, BEVs penetrate the epithelial layer to reach Peyer’s patch (Al-Nedawi et al., 2015). BEVs released by *Bacteroides fragilis* can also induce Th17 cell proliferation (Shen et al., 2012). In summary, BEVs derived from certain bacterial types can control inflammation and regulate immunity.

### BEVs Affect Intestinal Barrier Function

Impaired intestinal barrier function significantly affects the occurrence and progression of MASLD (Zhang et al., 2022). Disrupted tight junctions (Tj) between intestinal epithelial cells lead to barrier destruction and intestinal leakage; therefore, intestinal endotoxins and pathogens can more easily pass through the intestinal epithelium and enter the lamina propria, affecting the health of the host and inducing metabolic diseases such as MASLD. The intestinal microbiota and their metabolites, particularly BEVs, play key roles in this process. Recent studies have found a significant increase in the plasma levels of LPS^+^ BEVs and a significant increase in pathogen-associated molecular patterns in patients diagnosed with intestinal barrier disorders (Tulkens et al., 2020). BEVs derived from *Fusobacterium nucleatum* cause intestinal epithelial apoptosis by activating receptor-interacting protein kinases 1/3 (RIPK1/RIPK3), thereby inducing intestinal epithelial oxidative stress and increasing intestinal barrier permeability (Liu et al., 2021). The BEVs of *Campylobacter jejuni* exhibit proteolytic activity. Upon co-incubation with intestinal epithelial cells, BEVs cleave E-cadherin and clostridial proteins (Elmi et al., 2016). Cytotoxin-associated antigen A (CagA) is a carcinogenic factor produced by *Helicobacter pylori* (Backert and Blaser, 2016). The BEVs of *Helicobacter pylori* carrying CagA are localized to Tj zonula occludens-1 (ZO-1) and lead to the binding of the histone H1 subtype to ATP, which in turn affects host gene transcription (Turkina et al., 2015). Intestinal epithelial damage and mucosal inflammation lead to the translocation of intestinal bacteria and bacterial products (Xin et al., 2014), causing Kupffer cell activation. The increased release of inflammatory factors induces severe steatosis, inflammation, and fibrosis (Liu et al., 2023, Rahman et al., 2016). However, several studies have found that BEVs derived from probiotics benefit the intestinal barrier. The BEVs of EcN and ECOR63, a commensal bacterium of EcN, enhance the epithelial barrier function by upregulating the expression of intestinal epithelial Tj proteins, including ZO-1 and the transmembrane protein claudin-14 (Alvarez et al., 2016). These BEVs inhibit pathogenic *Escherichia coli*-induced infection by activating the transcriptional compensation of claudin-14 and occludin (Alvarez et al., 2019). The BEVs of *Akkermansia muciniphila* improve obesity, T2D barrier integrity, and metabolic function by increasing the expression of occludin and activating the adenosine monophosphate (AMP)-activated protein kinase pathways (Chelakkot et al., 2018). The BEVs of *Akkermansia muciniphila* mediate immune regulation of the colon and intestinal barrier functions (Kang et al., 2013). Additionally, BEVs from *Lactobacillus rhamnosus* GG increased the activity of intestinal AhR/IL-22/regenerating islet-derived protein 3γ (Reg3γ) signaling pathway, increased the expression of intestinal Tj protein, led to the reduction of bacterial translocation in the liver, reduced liver fat accumulation, and improved liver function (Gu et al., 2021). This indicates that the beneficial or harmful effects of BEVs on the intestinal barrier may vary depending on the strain.

## EVS WITH THERAPEUTIC POTENTIAL FOR MASLD

The discovery and research of various EVs provide novel strategies for the treatment of MASLD. We systematically analyze these EVs below.

### BEVs From Bacteria

Like other EVs, BEVs are lipid bilayer membrane vesicles containing various proteins, endotoxins, lipids, and nucleic acids. EVs isolated from feces can protect their microRNA (miRNA) from RNase degradation for 90 min. The miRNA of EVs extracted from the human colorectal cancer cell line HT-29 begins to degrade significantly after RNase treatment for 30 min, whereas the free miRNA extracted from feces degrades rapidly (Koga et al., 2011). Treatment with RNase or Triton X-100 alone resulted in little or no degradation of the total OMV RNA. The total RNA concentration decreased significantly after the double treatment (Bittel et al., 2021). OMVs protect the activity of their cargo under high-temperature conditions, lyophilization, and iterative freeze-thaw cycles (Alves et al., 2016). Therefore, BEVs have remarkable stability, ensuring that their active substances are free from decomposition during long-distance delivery (GillRahman et al., 2016; , 2019). BEVs have good cell permeability and can enter recipient cells in various ways, including fusion, receptor-ligand binding, and phagocytosis. BEVs can spontaneously rupture and release their contents and diffuse into target cells; BEVs can also attach to target cells, lyse, fuse, or be internalized into cells (Kulp and Kuehn, 2010); BEVs can also enter cells through lipid rafts (O’donoghue and Krachler, 2016). The BEVs of Nissle 1917 showed good compatibility with bone marrow mesenchymal stem cells (Liu et al., 2024). BEVs from *Akkermansia muciniphila* are ingested by colon adenocarcinoma cells (Fábrega et al., 2016). However, immunogenicity is one of the main limitations of BEVs. BEVs from *Bacteroides fragilis* (Shen et al., 2012), *Staphylococcus aureus* (Bitto et al., 2021), *Bacteroides thetaiotaomicron* (Fonseca et al., 2022), and *Legionella pneumophila* (Jung et al., 2016) stimulate the release of inflammatory factors via the TLR-2 signaling pathway. Additionally, many BEVs cause inflammatory reactions through other channels, including caspase-11 activation (Elizagaray et al., 2020), TLR-9 (Rodriguez and Kuehn, 2020), and TLR-3 (Bitto et al., 2021), among others.

Most studies have shown that BEVs mediate metabolic disorders, inflammation, and intestinal damage, thereby inducing the pathological processes of MASLD. However, there is evidence that BEVs derived from some bacterial species can improve the metabolic state of the host. For instance, the administration of BEVs from *Akkermansia muciniphila* via oral gavage in HFD-fed mice significantly improved IR and reduced body weight (Chelakkot et al., 2018). This conclusion was confirmed by a subsequent study (Ashrafian et al., 2019), demonstrating that *Akkermansia muciniphila* BEV treatment enhanced adipose tissue β-oxidation, concurrently lowering body weight, blood glucose, and serum cholesterol levels in HFD-fed mice while alleviating adipose tissue inflammation. Mechanistically, BEVs from *Lactobacillus rhamnosus* GG exert hepatoprotective effects by suppressing miR-194 expression in the intestinal epithelium, thereby activating the farnesoid X receptor signaling pathway, reducing hepatic bile acid synthesis, and attenuating lipid accumulation in the liver (Jiang, 2022). Furthermore, *Lactobacillus rhamnosus* GG-derived BEVs enhanced intestinal AhR-dependent IL-22/Reg3γ signaling, which upregulated Tj proteins (eg, occludin and ZO-1) in the gut epithelium. This reinforcement of the intestinal barrier integrity reduces microbial translocation to the liver, ultimately attenuating hepatic lipid deposition and restoring liver function (Gu et al., 2021). In an in vitro coculture of HepG2 cells with *Lactobacillus paracasei* or *Lactobacillus casei*, their BEVs exhibited no cytotoxic effects. Notably, these probiotic coculture systems significantly enhanced hepatic synthetic functions, as evidenced by elevated urea production and increased albumin synthesis levels (Kang et al., 2023). Existing evidence suggests that probiotic-derived BEVs have the potential to treat MASLD (Ashrafian et al., 2019, Chelakkot et al., 2018, Gu et al., 2021, Jiang, 2022, Kang et al., 2023). However, only a few relevant studies have been conducted, and the efficacy mechanisms remain unclear.

### EVs From Other Sources

Apart from BEVs, EVs from other sources have also been extensively studied. Stem cell-derived EVs show therapeutic potential in MASLD, particularly mesenchymal stem cell EVs (MSC-EVs). Adipose-derived MSC-EVs transfer miR-223-3p to hepatocytes, suppressing E2F1 to mitigate lipid accumulation and fibrosis in palmitic acid-induced hepatocytes and HFD mice (Niu et al., 2022). EVs derived from beige adipose stem cells increase the expression of genes related to adipose tissue browning and function, increase insulin sensitivity, and improve HFD-induced liver fat accumulation (Jung et al., 2020). MSC-EVs derived from human umbilical cord can enhance the expression of glucose-6-phosphatase, phosphoenolpyruvate carboxykinase, fatty acid synthetase, and sterol regulatory element-binding protein 1c (SREBP1c) through miR-627-5p; miR-627-5p targets fat mass and obesity-associated gene to regulate glucose and lipid metabolism, thereby reducing the liver weight and liver index (Cheng et al., 2021). MSC-EVs inhibit SREBP1c and enhance the expression of peroxisome proliferator-activated receptor α by transferring calcium-/calmodulin-dependent protein kinase 1 to hepatocytes to regulate lipid oxidation and synthesis and reduce lipid accumulation (Yang et al., 2023). EVs generated by bone marrow stem cells inhibit caspase-2 by upregulating miR-96-5p, thus inhibiting fat synthesis and stimulating fatty acid oxidation in HFD-fed rats, ultimately alleviating hepatic steatosis (El-Derany and AbdelHamid, 2021).

Immune cells play an important role in disease resistance, and their EVs exert positive effects in preventing hepatic steatosis. EVs derived from neutrophils are rich in miR-223, which can be selectively ingested by hepatocytes and play a role in inhibiting liver inflammation and fibrosis, depending on the expression of apolipoprotein E and low-density lipoprotein receptor in hepatocytes (He et al., 2021). Neutrophil-derived EVs may inhibit NLRP3 through miR-223 and promote the transformation of hepatic macrophages from a proinflammatory phenotype to a restorative phenotype (Calvente et al., 2019). EVs produced by Kupffer cells loaded with miR-690 target liver macrophages, reduce inflammation and fibrosis of HSCs, and inhibit fatty acid synthesis in hepatocytes (Gao et al., 2022). EVs derived from macrophages inhibit HSC activation, thereby attenuating fibrosis in MASH, which may be related to the targeted downregulation of calmodulin-regulated spectrin-associated protein 1 by miR-411-5p encapsulated in EVs (Wan et al., 2022).

Plant-derived exosome-like nanoparticles reshape the intestinal microbiota, improve inflammation, regulate metabolic disorders, and are expected to be an effective strategy for the treatment of MASLD. After HFD mice were fed garlic-derived exosome-like nanoparticles (GaEN), the number of *Akkermansia muciniphila* increased by approximately 40 times, and the release of BEVs also increased compared to that in the control group (Sundaram et al., 2024). After incubation with GaEN, the release of OMV from *Akkermansia muciniphila* increased, and the OMV increased the expression of the IRS1/IRS2 signaling pathway by increasing circulating GLP-1 and stimulating the GLP-1 receptor, thus promoting glucose uptake (Sundaram et al., 2024). The same team also reported that GaEN reduced IR and reversed HFD-induced obesity by inhibiting the indoleamine 2,3-dioxygenase1/AhR pathway (Sundaram et al., 2022). Exosome-like nanoparticles from pomegranate enter HepG2 cells and alleviate palmitic acid-induced lipid accumulation by improving mitochondrial dysfunction. Oral pomegranate in mice fed with HFD can improve oxidative stress injury and fatty degeneration of the liver by reversing the decrease in the SIRT3/superoxide dismutase 2 signaling pathway (Hou et al., 2023). Furthermore, ginger-derived exosome-like nanoparticles reduced the inactivation of Foxa2 by inhibiting Akt1-mediated phosphorylation and alleviating intestinal epithelial cell-mediated IR induced by HFD. Foxa2 can activate lipid mobilization under normal circumstances, but is inactivated in IR (Kumar et al., 2022). After oral administration of exosome-like nanoparticles from kidney beans, the blood sugar, blood lipid, body weight, and liver weight of obese rats significantly decreased, which may be related to beneficial changes, such as intestinal microbiota remodeling and increased SCFAs (Pang et al., 2024).

In summary, EVs from different sources may improve the MASLD in different ways.

## CONCLUSION AND PERSPECTIVES

MASLD is a leading cause of chronic liver disease, placing a significant medical and economic burden on individuals and society. Therefore, it is of great clinical value to explore the pathophysiological mechanisms of MASLD to develop targeted treatments. BEVs play an important role in the biological processes involved in MASLD. Previous studies have noted the potential hazards of BEVs in inducing diseases, and recent studies have found that BEVs produced by some intestinal bacteria have beneficial regulatory effects. BEVs are closely related to the state of their parent cells and are differentially expressed in healthy individuals and patients with MASLD. BEVs have significant stability and cell permeability; therefore, they are potential novel therapeutic targets. Improvement in MASLD by intestinal microbiota has been confirmed; however, research related to BEVs is still relatively lacking.

Many challenges still need to be overcome before the clinical transformation of BEVs. First, the complex and volatile internal composition of BEVs remains poorly understood, and potential molecular mechanisms have not been effectively explored. Second, BEVs are defined by size and concentration, but current isolation methods face high cost, cumbersome workflows, and poor scalability. Third, the immunogenicity of BEVs may be one of the main limitations in their research and application. Finally, research on BEVs is mostly at the laboratory stage, and BEVs still face an arduous battle for clinical application. Fortunately, many studies have found that EVs from stem cells, immune cells, and plants have therapeutic effects on MASLD, and that probiotic BEVs also have beneficial effects, providing a new therapeutic strategy for MASLD treatment.

## ORCID

Li-Na Qin: 0000-0002-4015-3023

Yun-Feng Yu: 0000-0002-7309-5608

Lie Ma: 0000-0002-6137-9822

Rong Yu: 0009-0005-0840-2797

## Funding and Support

This research was supported by a grant from the National Natural Science Foundation of China (U21A20411, 82074400), the Hunan Provincial Natural Science Foundation Innovation Research Group Project (2024JJ1007), and the Hunan University of Chinese Medicine Disciplinary Construction “Revealing the List and Appointing Leaders” Project (22JBZ002).

## CRediT Authorship Contribution Statement

**Qin Li-Na** and **Yu Yun-Feng**: Writing—original draft, Writing—review and editing, Formal analysis. **Ma Lie**: Writing—original draft, Software. **Yu Rong**: Writing—review and editing, Supervision.

## DECLARATION OF COMPETING INTERESTS

The authors declare that they have no known competing financial interests or personal relationships that could have appeared to influence the work reported in this paper.
